# MnOx-*Coffea arabica* Husk and *Catha edulis* Leftover Biochar Nanocomposites for Removal of Methylene Blue from Wastewater

**DOI:** 10.1155/2024/7585145

**Published:** 2024-02-23

**Authors:** Jemere Kochito, Abera Gure, Negera Abdissa, Tamene Tadesse Beyene, Olu Emmanuel Femi

**Affiliations:** ^1^Department of Chemistry, College of Natural Sciences, Jimma University, P.O. Box 378, Jimma, Ethiopia; ^2^Department of Chemistry, College of Natural and Computational Sciences, Wollega University, P.O. Box 395, Nekemte, Ethiopia; ^3^Department of Chemical Engineering, School of Material Science, Institute of Technology, Jimma University, P.O. Box 378, Jimma, Ethiopia

## Abstract

In this study, we investigated the use of manganese oxide-biochar nanocomposites (MnOx-BNC), synthesized from coffee husk (CH) and khat leftover (KL) for the removal of methylene blue (MB) from wastewater. Pristine biochars of each biomass (CH and KL) as well as their corresponding biochar-based nanocomposites were synthesized by pyrolyzing at 300°C for 1 h. The biochar-based nanocomposites were synthesized by pretreating 25 g of each biomass with 12.5 mmol of KMnO_4_. To assess the MB removal efficiency, we conducted preliminary tests using 0.2 g of each adsorbent, 20 mL of 20 mg·L^−1^ MB, pH 7.5, and shaking the mixture at 200 rpm and for 2 h at 25°C. The results showed that the pristine biochar of CH and KL removed 39.08% and 75.26% of MB from aqueous solutions, respectively. However, the MnOx-BNCs removed 99.27% with manganese oxide-coffee husk biochar nanocomposite (MnOx-CHBNC) and 98.20% with manganese oxide-khat leftover biochar nanocomposite (MnOx-KLBNC) of the MB, which are significantly higher than their corresponding pristine biochars. The adsorption process followed the Langmuir isotherm and a pseudo-second-order model, indicating favorable monolayer adsorption. The MnOx-CHBNC and MnOx-KLBNC demonstrated satisfactory removal efficiencies even after three and six cycles of reuse, respectively, indicating their potential effectiveness for alternative use in removing MB from wastewater.

## 1. Introduction

Dyes are commonly used to color products in heavy industries such as textiles, paper, and food processing [1]. Globally, more than 700,000 tons of dyes are produced each year, with approximately 100,000 different types of dyes being used in various industries [2]. Literature reports indicate that the discharge of dyes from industries contributes to 20% of water pollution [3, 4]. These dyes can have negative effects on aquatic ecosystems, the food chain, and public health [5]. Even at low concentrations, they reduce aesthetic value, hinder light penetration, and impact gas solubility for photosynthesis and respiration processes [6].

Methylene blue (MB) is an organic dye that is widely used to color paper, print cotton, and dye leather and plastics [7–9]. Prolonged exposure to MB can lead to tissue necrosis and cyanosis and pose threats to marine life [10]. It enters the ecosystem through the discharge of untreated or partially treated industrial effluents. Therefore, it is crucial to implement appropriate treatment procedures before releasing industrial effluents into the environment.

Various methods, including photocatalytic degradation [11, 12], ultrafiltration [7, 13], electrocoagulation [14], electrochemical degradation [15], chemical precipitation [16], and adsorption [17], have been used for dye removal from industrial effluents [18]. However, some of these methods have limitations such as high-energy consumption, cost, generation of toxic sludge, incomplete treatment, and significant reagent consumption [19, 20]. Among these methods, adsorption is a cost-effective, eco-friendly, and efficient solution for removing organic and inorganic pollutants [2].

The efficiency of the adsorption method primarily relies on the adsorbent materials used [21]. Activated carbon, natural clay minerals, synthetic inorganic materials, synthetic nanoparticles, and biomass have all been utilized as adsorbents to remove various contaminants from aqueous solutions [22]. Recently, researchers have been focusing on finding low-cost, efficient, and selective materials for the removal of toxic chemicals like dyes from wastewater [5]. While activated carbon is widely used for water treatment, its raw materials and preparation methods are expensive and labor-intensive [23]. Materials that are porous and possess a high surface area are known to have good adsorption capacity. Nowadays, scholars are exploring the use of activated biochar to overcome some of the drawbacks associated with activated carbon [24]. Biochar is a porous material produced through the pyrolysis of biomass at temperatures below 700°C under oxygen-limited conditions. However, pure biochar also has limited adsorption performance, so it needs to be modified by combining it with suitable nanomaterials [25].

Metallic oxide nanoparticles such as magnetic ferric oxide, manganese oxide, titanium oxide, and magnesium oxide which have high specific surface areas have been used in wastewater treatment [26]. However, nanoparticles aggregation presents a challenge, necessitating the use of supporting materials to enhance their stability and recyclability [3]. The biochar-based nanocomposite involves the use of a composite material that combines the advantages of biochar, such as porosity and a higher specific surface area, with nanomaterials [16].

Metal salts such as AlCl_3_, CaCl_2_, MgCl_2_, KMnO_4_, MnCl_2_, ZnCl_2_, and TiCl_4_ are commonly used to activate biochar, resulting in the formation of Al_2_O_3_, AlOOH, CaO, MgO, MnOx, ZnO, and TiO_2_ nanoparticles on the biochar surface [27]. Most biochar-based nanocomposites are synthesized by chemical activation using metallic ions. The synthesis can proceed in either a one-step or two-step modification process [16]. In the one-step process, both carbonization and activation are completed simultaneously in the presence of an activator. In the two-step process, the biomass feedstock is carbonized first, followed by activation using the appropriate salt. The metal ions used for activation are either attached to the surface or enter the interior of the biomass upon impregnation or dipping into salt solutions. After pyrolysis, the metal ions are transformed into nanometal oxide or metal hydroxide, and the biomass impregnated with metal ions becomes biochar-based nanocomposites [26].

Some reports show that different metallic oxide nanocomposites such as Fe_3_O_4_ nanocomposites of saw dust, rice husk, palm oil empty fruit bunch, spent coffee ground biochar, and activated carbon are employed to remove MB from aqueous solutions [5, 28–32]. In addition, KMnO_4_-activated sludge [33], pine [34], and MnO_2_ orange peel [35] biochar nanocomposites are used for MB removal. KMnO_4_ is a strong oxidizing agent that can be used for water disinfection and oxidation of toxic matter. It can undergo mild oxidation of biomass at room temperature [33]. The KMnO_4_-activated biochar production process offers several advantages. It has a shorter activation time at room temperature, a mild reaction with organic materials, and produces a biochar with less ash content. Additionally, it is more environmentally friendly compared to other activation processes. Because of these advantages, both manganese oxide-coffee husk and khat leftovers biochar nanocomposite (MnOx-CHBNC and MnOx-KLBNC) can be utilized as environmentally preferred alternatives for the removal of pollutants from the environment. However, to the best of our knowledge, no investigation has been reported regarding the utilization of MnOx-CHBNC and MnOx-KLBNC for the removal of MB from aqueous solutions.

In Ethiopia, there is high production and consumption of coffee (*Coffea arabica*) and khat (*Catha edulis*), which generate tons of biomass waste that pollutes the environment [36, 37]. The unaddressed disposal of CH and KL increases municipal waste, leading to higher transportation costs when taken to the disposal area [38]. The objective of this study was to evaluate the conversion of CH and KL into useful products, which offers dual advantages: removing toxic pollutants from wastewater and disposing of biomass waste from the environment. The MnO_X_-CHBNC and MnO_X_-KLBNC were synthesized, characterized, and evaluated for their MB removal efficiencies. The study also examined the effects of contact time, adsorbent dose, initial concentration, and pH on the adsorption efficiency of MB for the two adsorbents. Additionally, the study investigated the kinetics and adsorption isotherms and conducted desorption studies to assess the regeneration or reusability of the adsorbents.

## 2. Materials and Methods

### 2.1. Chemicals and Materials

Coffee husk samples were collected from coffee pulping industries in Mizan-Aman town, and khat leftovers were collected from Jimma town, both in Ethiopia. These two biomasses were chosen because they are easily accessible and make a significant contribution to the municipal solid waste problem, which has led to severe environmental pollution across the country.

Various chemicals and reagents were used in the study, including KMnO_4_ (99%, Finkem), MB; C_16_H_18_N_3_SCl (99%, NICE), HNO_3_ (69%, Qualikems Fine chemicals), ultrapure NaOH (99%, Merck), and NaCl (99.5%, Sigma-Aldrich).

The crystallinity of the materials was determined using an X-ray diffractometer (DRAWELL Artist of Science XRD-7000, Shanghai, China). The surface properties were analyzed using scanning electron microscopy (SEM FEI QUANTA 250, Romania). Fourier transform infrared (FTIR) spectroscopy (Spectrum 65 FTIR, PerkinElmer) was used to analyze the surface functional groups of the materials. A muffle furnace (DRAWELL Artist of Science Muffle Furnace 1000°C SX-4-10, Shanghai, China) was used for the pyrolysis process. Double beam UV-Vis spectroscopy (SPECORDR200 PLUS Analytik Jena, Japan) was used for MB analysis.

### 2.2. Preparation of MnO_X_-CHBNC and MnO_X_-KLBNC

Khat (*Catha edulis*) leftovers were sliced into small pieces and washed with distilled water. They were then dried at 105°C for 24 h, ground into powders, and preserved [29, 39]. Similarly, the coffee husk was cleaned, dried, ground, and preserved using the same procedures.

For the synthesis of biochar nanocomposite materials, 25 g of each biomass powder was separately immersed in a 300 mL solution containing various concentrations of KMnO_4_: 12.5, 25, 50, and 75 mmol [39–43]. After stirring for 1 h, the mixture was evaporated to dryness in an oven at 80°C until the weight of the mixture remained constant. The dried sample was transferred to a crucible, covered with aluminum foil, and placed into a muffle furnace initially heated at 110°C. The sample was placed at 110°C for 30 min and then heated with a heating ramp of 10°C min^−1^ until it reached 300°C. Finally, it was pyrolyzed at 300°C for 1 h. The synthesized metal oxide/hydroxide-biochar nanocomposites were then cooled to room temperature, ground, and sieved with mesh sizes of 0.1 mm-0.2 mm. The pristine biochar was also synthesized using 25 g of dried biomass powder. It was repeatedly washed with distilled water until the washout became clear,then oven-dried at 80°C and kept for further experiments. The same procedures were followed to synthesize the biochar nanocomposites of the specified metallic oxides at 400 and 500°C. The prepared biochar nanocomposites were labeled as MnO_X_-CHBNC_300_, MnO_X_-CHBNC_400_, MnO_X_-CHBNC_500_, MnO_X_-KLBNC_300_, MnO_X_-KLBNC_400_, and MnO_X_-KLBNC_500_.

### 2.3. Adsorbent Selection

Following the procedures mentioned in the previous section, we produced 30 different adsorbents. We then evaluated the synthesized materials to determine the most efficient adsorbent for removing MB from an aqueous solution. The preliminary evaluations were conducted to assess the efficiency of each adsorbent in removing MB. These evaluations were conducted using 0.2 g of each adsorbent and 20 mL of a 20 mg·L^−1^ MB solution at pH 7.5. The mixture was then shaken at 200 rpm for 2 h, following the experimental procedures reported by Giraldo and coworkers [44]. After shaking, the mixture was centrifuged at 5000 rpm for 10 min. The resulting supernatant was then transferred to a cuvette for UV-Vis analysis at *λ* = 665 nm. Each experiment was conducted in triplicate.

The removal efficiency of each adsorbent for MB was calculated using equation (1), and the dye adsorption capacity of the materials was determined using equation (2).

(1) $$\begin{array}{c} \%R=\frac{\left(C_{o}-C_{e}\right)}{C_{o}}\times 100, \end{array}$$

(2) $$\begin{array}{c} q_{e}=\frac{\left(C_{o}-C_{e}\right)V}{m}. \end{array}$$

*C*_*o*_ (mg·L^−1^) and *C*_*e*_ (mg·L^−1^) represent the initial and equilibrium concentrations of the adsorbate, respectively, *m* (g) is the mass of the adsorbent, and *V* (L) is the volume of the sample solution [45].

### 2.4. Adsorption Isotherm and Kinetics

Batch adsorption experiments were conducted using 20 mL aqueous samples containing different initial concentrations of MB ranging from 10 to 500 mg·L^−1^. To each dye solution, 0.2 g of MnO_X_-CHBNC_300_ was added at 25°C. Similarly, for other sets of dye solutions, 0.15 g of MnO_X_-KLBNC_300_ was added. The mixtures were then shaken at 200 rpm using a horizontal shaker for 2 h. Subsequently, the samples were filtered, and the equilibrium concentrations of MB in each solution were measured by UV-Vis spectrometry at 665 nm.

### 2.5. Regeneration Studies

The reusability of the adsorbents was evaluated by performing, adsorption-desorption for six cycles at 25°C following the experimental design reported by Păcurariu and coworkers [3]. Accordingly, 2 g of MnO_X_-CHBNC and 1.5 g of MnO_X_-KLBNC were separately dispersed in 200 mL of a 20 mgL^−1^ MB solution by shaking for 120 min at pH 7.5. The solutions were then centrifuged, and the concentrations of MB in the supernatant were analyzed. For desorption, 2 g of the MB-loaded adsorbents was dispersed in 50 mL of 50% ethanol at pH 6.5. The mixture was shaken for 120 min and then separated by filtration. After each cycle, the MnO_X_-CHBC and MnO_X_-KLBNC adsorbents were washed with distilled water, dried at 70℃ for 2 h, and reused for adsorption in the next cycle.

## 3. Results and Discussion

### 3.1. Adsorbent Selection

In this study, biochar-based nanocomposites were prepared by varying the pyrolysis temperature and the mass of the activating agent, KMnO_4_.  Table 1 presents the results of the preliminary experimental data for the selection of adsorbents for the removal of MB from an aqueous solution. The experiments showed that the different adsorbents synthesized in this study had varying efficiencies in removing MB from the aqueous solution. These differences can be attributed to the type of biomass, pyrolysis temperature, and the amount of activating agent used.

**Figure 1:**
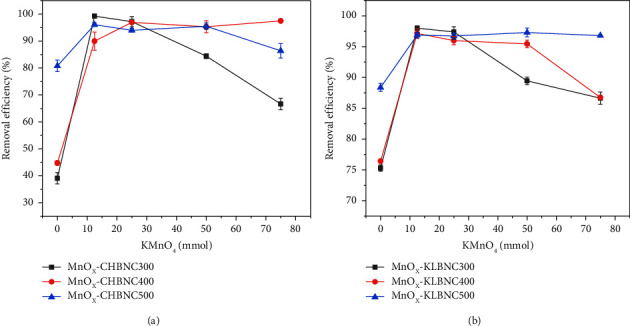
Pyrolysis temperature and biomass to activating agent (KMnO_4_) ratio evaluation: (a) MnO_X_-CHBNC and (b) MnO_X_-CHBNC. Experimental conditions: MB initial concentration, 20 mg/L; adsorbent dose, 0.2 g/L; pH, 7.5; temperature, 25°C; contact time, 120 min.

The effects of pyrolysis temperature and the activating agent-to-biomass ratio on the efficiency of MnO_X_-CHBNC and MnO_X_-KBNC for removing MB from an aqueous solution were investigated. Figures 1(a) and 1(b) show the effects of pyrolysis temperature and activating agent-to-biomass ratio on the efficiency of MnO_X_-CHBNC and MnO_X_-KBNC. The efficiency of MB removal from the aqueous solution increased as the pyrolysis temperature of pristine CHB and KLB increased. Yang and coworkers [46] reported that the pyrolysis temperature can influence both the yield and surface properties. They have found that higher pyrolysis temperatures lead to a decrease in the amount of biochar and acidic functional groups (-COOH and -OH), while alkaline functional groups, ash content, and pH increase. Therefore, the pyrolysis temperature can affect the adsorption efficiencies of MnO_X_-CHBNC and MnO_X_-KLBNC for MB. Treatment with KMnO_4_ significantly increased the adsorption efficiency from 39.08% to 99.26% for CHB and from 75.26% to 98.20% for KLB. The results also indicated that the amount of activating agent has an influence on the removal efficiency. In this study, the highest efficiency was observed when 25 g of each biomass was pretreated with 12.5 mmol of KMnO_4_ (2 : 1 g·mmol^−1^ ratio). Overall, the study revealed that MnO_X_-CHBNC and MnO_X_-KLBNC synthesized by pretreating 25 g of biomass with 12.5 mmol of KMnO_4_ and pyrolyzed at 300°C for 1 h exhibited better efficiency compared to other types of biochars synthesized in this study.

### 3.2. Adsorbent Characterization

**Figure 2:**
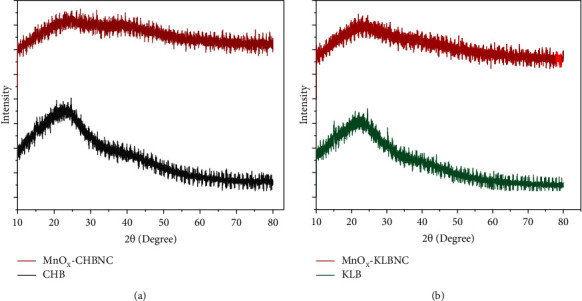
XRD patterns of (a) CHB and MnO_X_-CHBNC and (b) KLB and MnO_X_-KBNC.

Figures 2(a) and 2(b) show the XRD patterns of the pristine CHB and MnO_X_-CHBNC and KLB and MnO_X_-KLBNC, respectively. All adsorbents are composed of natural cellulose, lignin, and noncrystalline hemicelluloses. The diffraction peaks at 2*θ* = 16.1° and 22.4° can be assigned to natural cellulose, which is consistent with the findings of Baig and coworkers [47]. Reports indicate that the large d-spacing in the XRD peaks of biochar is due to the presence of unconverted cellulose and -OH, C=C, and C-O groups [48]. Crystalline materials generally exhibit sharper and more intense peaks compared to amorphous materials [49, 50]. The broadening of XRD peaks is primarily caused by particle size and lattice strain arrangement. Scattering of X-rays from the nonuniformly arranged surface materials and pores within the biochar results in broad peaks [30, 51]. Weak broad peaks at around 37.4° and 41.2° indicate the nonuniform distribution of MnO_2_ in the biochar nanocomposite which leads to X-ray scattering [52]. In addition, the porous nature of biochar allows its pores to trap X-rays. Therefore, the diffraction peaks in the XRD patterns of both adsorbents cannot be indexed as crystallized. Generally, the pristine biochars of MnO_X_-CHBNC and MnO_X_-KLBNC are all amorphous.

**Figure 3:**
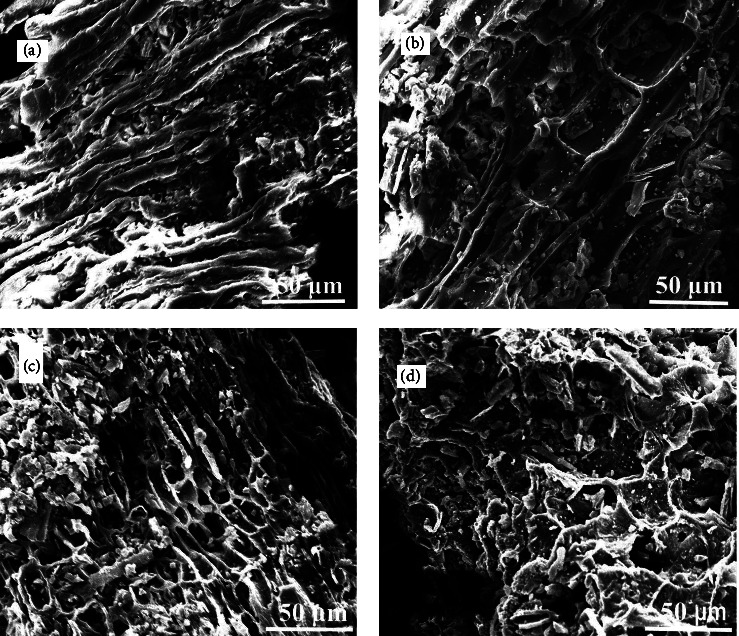
SEM images of pristine and activated coffee husk and khat leftover biochar. (a) CHB, (b) MnO_X_-CHBNC, (c) KLB, and (d) MnO_X_-KLBNC.

Figures 3(a)–3(d) show the SEM images of the activated and pristine biochar, CHB, and KLB. These images confirm that the synthesized biochars have amorphous and heterogeneous structures. Pores were observed in all biochars due to the escape of volatile substances and the formation of channel structures during pyrolysis [53]. The formation of porous structures is more prominent in activated biochars, as shown in Figures 3(b) and 3(d). According to the literature, activation increases porosity and enlarges the diameter of smaller pores created during pyrolysis [54].

**Figure 4:**
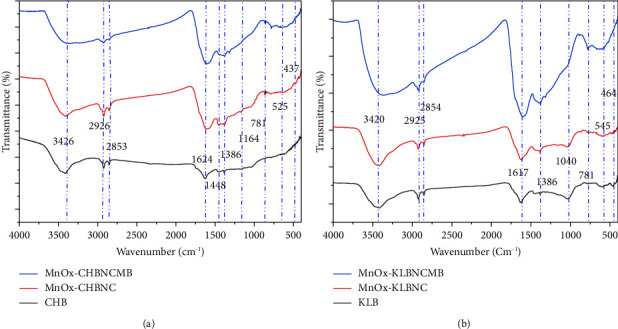
FTIR spectra of (a) CHB, MnO_X_-CHBNC, and MnO_X_-CHBNCMB and (b) KLB, MnO_X_-KLBNC, and MnO_X_-KLBNCMB.

The FTIR spectra of pristine and activated biochar are shown in Figure 4(a) for CHB and MnO_X_-CHBNC and in Figure 4(b) for KLB and MnO_X_-KLBNC. The spectra of both pristine and activated biochars showed the presence of functional groups such as O-H (3417–3426 cm^−1^), C-H (2853–2920 cm^−1^), C=C (1611–1622 cm^−1^), and C-O (1411–1466 cm^−1^) as reported in other literature [10, 17, 34, 55–57]. These functional groups may play a role in the adsorption of MB through electrostatic interaction [58]. The broad bands of O-H observed in the MnO_X_-CHB and MnO_X_-KLBNC spectra could be attributed to the additional sources of the OH group from the moisture [37].

Furthermore, there are broadening peaks with decreased intensity around 3425 cm^−1^ in the MB-adsorbed MnOx-CHBNC and MnOx-KLBNC FTIR spectra, as shown in Figures 4(a) and 4(b), which indicate the possibility of chemisorption occurring on the surface of biochar. This chemosorption leads to the formation of new compounds. The two main mechanisms of MB adsorption on biochar are the electrostatic attraction of cationic MB by the large number of OH groups in the solution at higher pH and the formation of hydrogen bonds between the oxygen present in MB and the OH groups of the biochars. Additionally, oxygen-containing functional groups form complexes with MB molecules through surface complexation resulting in MB adsorption on the adsorbents. After adsorption, the spectra at 1620 and 1382 cm^−1^ changed, with an increase in peak intensity, indicating an increase in the quantity of -C=C bonds caused by the cyclic alkene, most likely due to MB adsorption.

**Figure 5:**
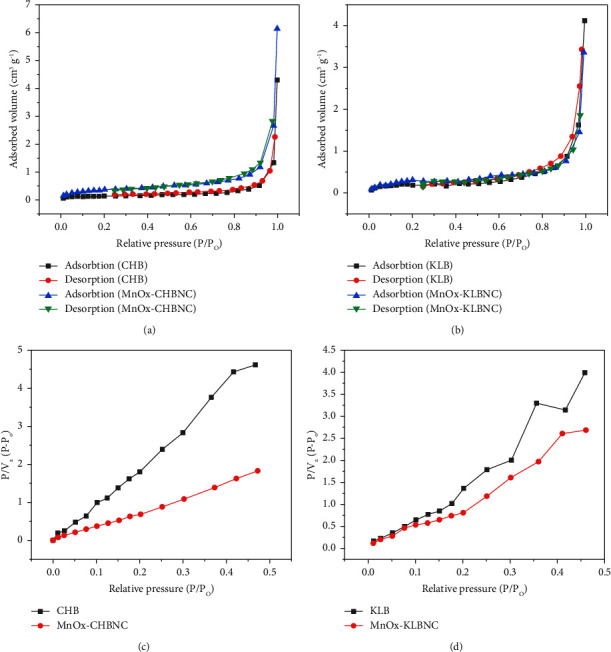
Adsorption-desorption isotherm of nitrogen on (a) CHB and MnO_X_-CHBNC and (b) KLB and MnO_X_-KLBNC, as well as BET analysis plot of (c) CHB and MnO_X_-CHBNC and (d) KLB and MnO_X_-KLBNC.

Figures 5(a)–5(d) present the adsorption-desorption isotherm and BET analysis plots for CHB, MnO_X_-CHBNC, KLB, and MnO_X_-KLBNC. Based on the results of the BET analysis, the specific surface area, pore volume, and pore size for CHB were reported as 0.519 m^2^·g^−1^, 0.004 cm^3^·g^−1^, and 32.804 nm, respectively. For MnO_X_-CHBNC, the values were 1.289 m^2^·g^−1^, 0.006 cm^3^·g^−1^, and 21.218 nm. The values for KLB were 0.826 m^2^·g^−1^, 0.005 cm^3^·g^−1^, and 27.626 nm, while for MnO_X_-KLBNC, they were 1.03 m^2^·g^−1^, 0.006 cm^3^·g^−1^, and 19.511 nm. The results revealed that MnO_X_-CHBNC and MnO_X_-KLBNC have higher specific areas, total pore volumes, and smaller pore sizes than their pristine biochars. In addition, according to IUPAC, the adsorption-desorption isotherm and BET analysis showed that the adsorbents exhibited mesoporous structures [31].

### 3.3. Batch Adsorption Studies

Out of the various adsorbents prepared in this study, MnO_X_-CHBNC and MnO_X_-KLBNC were chosen based on the results of the preliminary adsorbent selection experiment conducted in Section 2.3. The discussion of this selection is given, along with Table 1 and Figure 1. The parameters that can affect the adsorption efficiency of MnO_X_-CHBNC and MnO_X_-KLBNC were investigated at a constant temperature of 25°C.

#### 3.3.1. Point of Zero Charge

**Figure 6:**
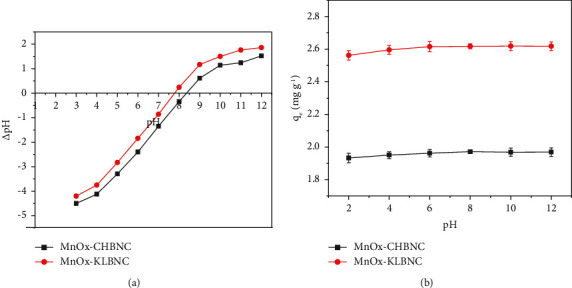
(a) Determination of pH_PZC_ and (b) the effect of pH.

The point of zero charge (pH_PZC_) of an adsorbent depends on the chemical and electronic properties of the functional groups on its surface. Figures 6(a) and 6(b) show the results of pH_PZC_ of MnO_X_-CHBNC and MnO_X_-KLBNC as well as the effect of pH on the adsorption process. The pH_PZC_ values were approximated at pH 7.82 for MnO_X_-KLBNC and 8.43 for MnO_X_-CHBNC. Therefore, pH values should be maintained above these values to ensure that the negatively charged surfaces favor adsorption through electrostatic attraction between the adsorbents and the cation (MB).

#### 3.3.2. Effect of pH

The pH of the solution affects the adsorption processes because it can change the surface charge of the adsorbent and the ionizable organic dye molecules [51]. The pH also determines the competition between cationic dyes and the adsorbent as well as the presence of extra OH^−^/H^+^ ions in the solution. As a result, the adsorption capacity of MB fluctuates.

In this study, the effect of pH was evaluated from 3 to 12 by adjusting it to the desired value using 0.10 M HNO_3_ and 0.10 M NaOH solutions.  Figure 6(b) shows the impact of the solution pH on the MB adsorption capacity of MnO_X_-CHBNC and MnO_X_-KLBNC. The initial MB concentration was 20 mg·L^−1^, with 0.2 g of MnO_X_-CHBNC and 0.15 g of MnO_X_-KLBN used as adsorbents. The contact times were 60 min for MnO_X_-CHBNC and 80 min for MnO_X_-KLBNC. The results showed that the adsorption capacity of MnO_X_-CHBNC increased from 1.93 to 1.97 mg·g^−1^ in the pH range of 2–8, while MnO_X_-KLBNC increased from 2.56 to 2.61 mg·g^−1^ in the pH range of 2–6. This can be attributed to the strong electrostatic repulsion generated by cationic MB dye molecules on the surface of biochar, which contain high concentration of H^+^ ions in an acidic environment. In addition, as mentioned by Islam and coworkers [52], the presence of the OH group on an adsorbent surface triggers protonation of the OH groups and creates competition between H^+^ ions and dye molecules for binding with active sites, resulting in low uptake of sorbate molecules.

Generally, both adsorbents showed significant differences between the lowest adsorption capacities at pH 2 and the maximum adsorption capacities at pH 12. The minimum removal efficiency remained above 96%. This indicates that the adsorbents can act as a buffer system, capable of resisting pH changes. Therefore, MnO_X_-CHBNC and MnO_X_-KLBNC can effectively remove MB in both acidic and alkaline media.

#### 3.3.3. Effect of Contact Time

**Figure 7:**
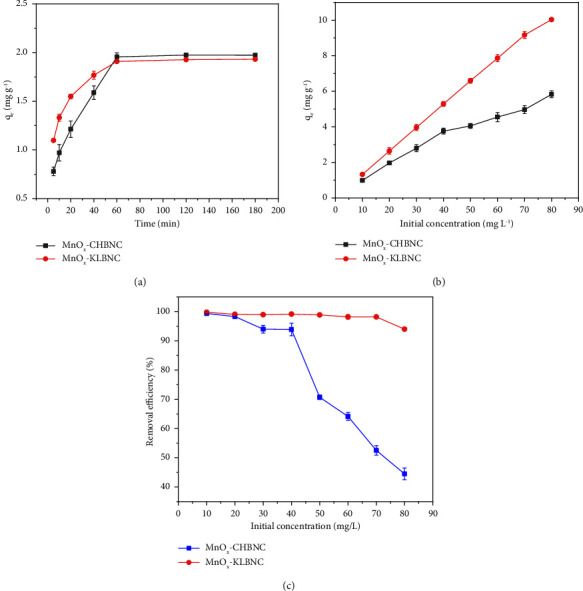
(a) The effect of contact time on MB adsorption capacity, (b) the effect of initial concentration on MB adsorption capacity, and (c) the effect of initial concentration on MB removal efficiency.

Contact time is one of the factors that affect the adsorption efficiency. In this study, the effect of time was investigated over the range of 5–180 min. The dosage used was 0.2 g, the pH was 7.5, and the initial concentration was 20 mg·L^−1^.  Figure 7(a) illustrates that the adsorption of MB was initially rapid during the early stage, but it gradually slowed down after 60 min. After 60 min, the adsorption rate remained relatively constant. The initial rapid adsorption rate can be attributed to the availability of a sufficient adsorption surface, which then decreases over time until equilibrium is attained. The presence of repulsive forces between the solute molecules adsorbed on the solid and the bulk phase creates obstacles for continuous adsorption on the remaining adsorption sites [53]. Kumar and coworkers also reported that the removal efficiency of alkaline-treated banana stem biochar for MB dye increases with longer contact time [58].

#### 3.3.4. Effect of Initial Concentration

The effect of the initial concentrations of MB was studied, ranging from 10 to 80 mg·L^−1^. The results indicate that as the initial concentration of MB increased, the adsorption capacity also increased (Figure 7(b)). The adsorption capacity of MnO_X_-CHBNC changed from 0.98 to 5.83 mg·g^−1^, while MnO_X_-KLBNC changed from 1.32 to 10.03 mg·g^−1^.

In general, molecules tend to adsorb more readily at higher initial concentrations due to the presence of a greater driving force required for the mass transfer of dye molecules. In addition, higher initial concentrations required a longer equilibrium time. This is because, during the final stage of adsorption, most of the sorbate molecules diffuse into the porous structure of the adsorbent as the adsorbent surface becomes saturated. These results are consistent with a study on the removal of MB by mangosteen peel biochar prepared via hydrothermal carbonization for methylene blue removal [54]. Therefore, an initial concentration of 20 mg·L^−1^ was chosen as the optimal concentration, at which MnOx-CHBNC and MnOx-KLBNC removed approximately 99.27% and 98.20% of MB, respectively.

Furthermore, Figure 7(c) demonstrates that under the optimal conditions, 0.2 g of MnOx-CHBNC can remove 93.87% of MB from a 20 mL solution with a concentration of 40 mg/L, while 0.15 g of MnOx-KLBNC can remove 94.00% of MB from a 20 mL aqueous solution with a concentration of 80 mg/L.

#### 3.3.5. Effect of Adsorbent Dose

**Figure 8:**
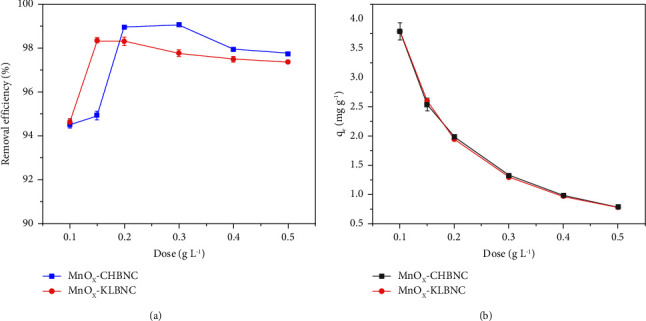
Effect of adsorbent dose on (a) removal efficiency and (b) adsorption capacity.

Figure 8(a) shows the variation in the removal efficiency of MB at different doses of MnO_X_-CHBNC and MnO_X_-KLBNC. The effects of adsorbent dosage were evaluated within the range of 0.1–0.5 g for both MnO_X_-CHBNC and MnO_X_-KLBNC, while keeping other parameters constant (pH 7.5, initial concentration 20 mg·L^−1^, and contact time 60 min). As shown in Figure 8(a), increasing the dose of MnO_X_-CHBNC from 0.1 g to 0.2 g resulted in a 4.34% increase in MB removal efficiency. Similarly, increasing the dosage of MnO_X_-KBNC from 0.1 g to 0.15 g led to 2.83% increase in removal efficiency. These improvements can be attributed to the increased number of available adsorption sites, which agrees with the findings of a study reported by Le and coworkers [55]. However, the adsorption of MB only slightly increased when the dose exceeded 0.15 g for MnO_X_-KLBNC and 0.2 g for MnO_X_-CHBNC, as the adsorbent surface eventually reaches a saturation state. Notably, when the dose of MnO_X_-CHBNC exceeded 0.5 g, the removal efficiency of MB decreased to 97.74%. This decrease may be attributed to the aggregation of adsorbents, which hinders the accessibility of binding sites and alters the viscosity of the solution, preventing the free movement of MB molecules [56]. Based on these results, further studies were conducted using 0.15 g of MnOx-KLBNC and 0.2 g of MnOx-CHBNC.

On the other hand, the adsorbent dose had a negative impact on the adsorption capacity (Figure 8(b)). The value of *q*_*e*_ decreased rapidly when the dosage of MnO_X_-CHBNC and MnO_X_-KLBNC increased from 0.1 to 0.5 g·L^−1^. Subsequently, the slope of the adsorption capacity curves decreased because at low adsorbent amounts, the active adsorption sites quickly combine with the adsorbates and approach saturation. When the amount of adsorbent exceeds a certain value, the increasing adsorption sites fail to come into contact with adsorbate molecules [57]. In addition, as the number of adsorbents increases, they tend to aggregate, resulting in a reduction in the specific surface area of the sorbents [58].

#### 3.3.6. Adsorption Isotherm and Kinetics Model

Adsorption isotherms relate the concentration of the adsorbate and the adsorption capacity at a specific dose of adsorbent and temperature [2]. Analyzing these isotherms helps in understanding the mechanisms of adsorption, which depend on factors such as surface polarity, surface area, and porosity. The linear forms of the Langmuir and Freundlich isotherm models can be used to quantify the equilibrium adsorption data (equations (3) and (4)) [59]. The Langmuir isotherm (equation (3)) describes adsorption on a surface with uniform active sites, forming a monolayer. The adsorption equilibrium was studied by fitting the experimental data to the linear equations of the Langmuir and Freundlich isotherm models [1].

(3) $$\begin{array}{c} \text{Langmuir isotherm model}:\frac{C_{e}}{q_{e}}=\frac{1}{K_{L}q_{m}}+\frac{C_{e}}{q_{m}}, \end{array}$$

(4) $$\begin{array}{c} \text{Freundlich isotherm model}:\log\;q_{e}=\log\;K_{F}+\frac{1}{n}\log\;C_{e}, \end{array}$$

where *q*_*e*_ (mg·g^−1^) is the amount of MB adsorbed, *C*_*e*_ (mg·L^−1^) is the adsorbate concentration in the solution at equilibrium, *K*_*L*_ is the Langmuir adsorption constant, and *q*_*m*_ (mg·g^−1^) is the maximum adsorption capacity for monolayer formation on the adsorbent [60]. The value of *K*_*F*_ is the adsorption or distribution coefficient, which represents the number of ions adsorbed onto the beads. The value of 1/*n* indicates surface heterogeneity; as its value gets closer to zero, the surface becomes more heterogeneous [61]. A fundamental characteristic of the Langmuir isotherm is to predict the affinity between adsorbate and sorbent using a dimensionless constant, known as the separation factor *R*_*L*_, which can be calculated from the following equation:

(5) $$\begin{array}{c} R_{L}=\frac{1}{1+K_{L}C_{o}}, \end{array}$$

where *C*_*o*_ (mg·L^−1^) is the adsorbate initial concentration. The value of *R*_*L*_ ranges between 0 and 1 for favorable adsorption, while *R*_*L*_ > 1 represents unfavorable adsorption, *R*_*L*_=1 represents linear adsorption, and *R*_*L*_=0 represents irreversible adsorption processes [61].

**Figure 9:**
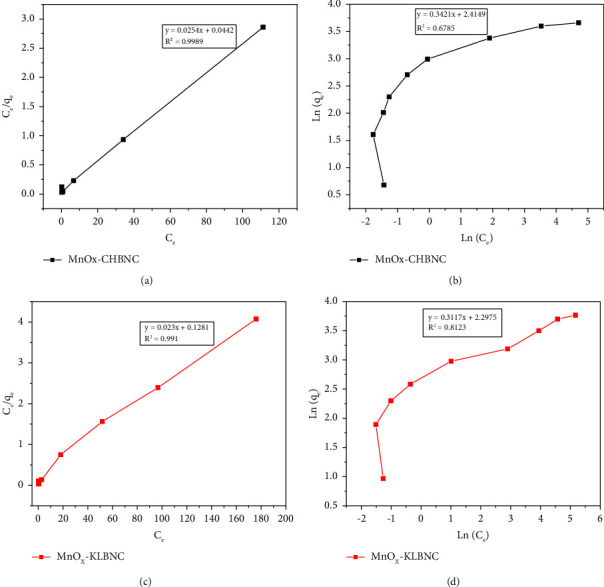
(a) Langmuir isotherm for MnO_X_-CHBNC, (b) Freundlich isotherm MnO_X_-CHBNC, (c) Langmuir isotherm for MnO_X_-KLBNC, and (d) Freundlich isotherm MnO_X_-KLBNC.

The Langmuir and Freundlich isotherm parameters were investigated using the initial concentrations ranging from 20 to 500 mg·L^−1^, and their results are presented in Figures 9(a)–9(d).

The Langmuir and Freundlich isotherm parameters and related correlation coefficients (Figures 9(a)–9(d)) are summarized in Table 1. A higher correlation coefficient (*R*^2^) indicates greater applicability of the Langmuir model with *R*^2^=0.999 for MnO_X_-CHBNC and *R*^2^=0.991 for MnO_X_-KLBNC, demonstrating the monolayer adsorption on a specific site of a homogeneous surface of the adsorbent [4]. The Langmuir isotherm predicts that the adsorption energy is uniform on the adsorbent surface and no interaction exists between the adsorbed molecules [10]. The low separation factor values (*R*_*L*_=0.080 for MnO_X_-CHBNC and *R*_*L*_=0.048 for MnO_X_-KLBNC) imply a favorable physical adsorption process. The Freundlich isotherm demonstrates multilayered adsorption for heterogeneous surfaces or surface-supporting sites of different affinities [57]. The calculated value of n falling in the range of 0 to 1 indicates favorable sorption. Furthermore, the Langmuir isotherm model has a higher regression coefficient *R*^2^ than the Freundlich model (Table 2), showing that the Langmuir model provides a better description. These results suggest monolayer adsorption of MB on the surface of MnO_X_-CHBNC and MnO_X_-KLBNC.

In terms of the nonlinearity of the Langmuir and Freundlich isotherms, Figure 9 also shows that the adsorbed amount of MB on both MnOx-CHBNC and MnOx-KLBNC increases with increasing concentration but not linearly. Specifically, Figures 9(b) and 9(c) show that the adsorption capacity will reach a plateau (saturation) at higher concentrations, especially for MnOx-CHBNC [62]. This suggests that there are a limited number of binding sites available on the adsorbent surface, which are eventually occupied, and there is no binding interaction between the MB molecules to form a multilayer. This is consistent with the monolayer adsorption of the Langmuir isotherm model, which is common nonlinear behavior [63, 64]. The nonlinearity behavior suggests that the adsorption process is not solely a physical interaction between the MB and the adsorbent surface; there might be chemical interactions involved. MnOx-KBNC appears to have a higher capacity for MB than MnOx-CHBNC at different initial concentrations.

Kinetic studies were conducted using 20.0 mL of MB solution with an initial concentration of 20 mg·L^−1^, pH 8.0, 0.2 g of MnO_X_-CHBNC, and 0.15 g of MnO_X_-KLBNC. The mixture was shaken for various time intervals (5, 10, 20, 30, 40, 60, 80, 120, 180, and 240 min) at 200 rpm and 25°C, following the previously reported procedure [54]. Afterwards, the solutions were centrifuged, and the concentrations of MB in the supernatant were determined. The amount of MB adsorbed onto MnO_X_-CHBNC and MnO_X_-KLBNC at time *t* (*q*_*t*_) was calculated using the following equation:

(6) $$\begin{array}{c} q_{t}=\frac{\left(C_{o}-C_{t}\right)V}{m}, \end{array}$$

where *C*_*o*_ is the initial concentration (mg·L^−1^), *C*_*t*_ is the concentration at time *t* (mg·L^−1^), *V* is the volume (L), and *m* is the mass of adsorbent (g). Two adsorption kinetics models (pseudo-first-order and pseudo-second-order) were employed. The pseudo-first-order adsorption kinetics model used the following equation:

(7) $$\begin{array}{c} \ln\left(q_{e}-q_{t}\right)=\ln\left(q_{e}\right)-K_{1}t, \end{array}$$

where *q*_*e*_ and *q*_*t*_ are the amounts of MB adsorbed (mg·g^−1^) at equilibrium and time *t* (min), respectively, and *K*_1_ is the rate constant for the pseudo-first-order kinetics model. The pseudo-second-order kinetics model used the following equation

(8) $$\begin{array}{c} \frac{t}{q_{t}}=\frac{1}{{{K_{2}}_{\text{qt}}}^{2}}+\frac{t}{q_{e}}, \end{array}$$

where *K*_2_ is the rate constant for the pseudo-second-order kinetic model of adsorption. The adsorption parameters, including *R*^2^ and other constants, were calculated for both models and are listed in Table 3. The results showed that the adsorption mechanisms were better represented by the pseudo-second-order model.

The maximum MB adsorption capacities of various adsorbents are listed in Table 4. The adsorption capacities achieved by the manganese oxide-biochar nanocomposites prepared in this study were higher than those reported in previous studies for certain adsorbents. This indicates that MnO_X_-CHBNC and MnO_X_-KLBNC are effective in removing MB from aqueous solutions. Furthermore, the production of biochar nanocomposites using CH and KL provides a valuable method for eliminating potential pollutants and creating value-added treatment products. CH and KL are low-cost biomass options for biochar production, making this approach suitable for resource recovery and environmental protection. The KMnO_4_-activated biochar production process offers several advantages, including a shorter activation time at room temperature, a mild reaction with organic materials, the formation of biochar with lower ash content, and greater environmental friendliness compared to other activation processes. Therefore, despite their slightly lower removal efficiencies compared to other options, MnOx-CHBNC and MnOx-KLBNC are more environmentally preferable to be used for the removal of MB and other related organic pollutants from aqueous solutions.

### 3.4. Regeneration Studies

**Figure 10:**
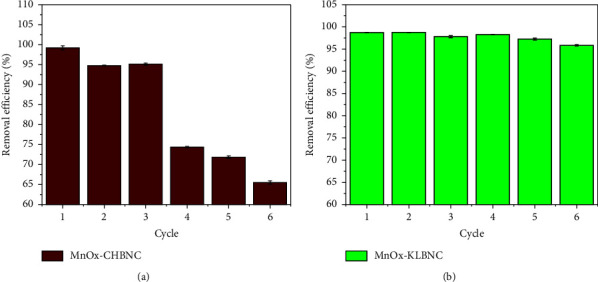
Regeneration of (a) MnO_X_-CHBNC and (b) MnO_X_-KLBNC for the removal of MB from aqueous solution.

The results revealed that one advantage of the proposed biochar nanocomposite adsorbents is their easy separation from soluble waste and reusability. To investigate the reusability, 2 g of MnOx-CHBNC and 1.5 g of MnOx-KLBNC adsorbent were separately added to a 200 mL solution containing 20 mg·L^−1^ of MB. The study results are shown in Figures 10(a) and 10(b). The findings demonstrated that the relative adsorption efficiency of MnOx-CHBNC significantly decreased after three cycles, while the relative adsorption efficiency of MnOx-KLBNC showed negligible decrease up to six cycles, indicating its better reusability compared to the other adsorbent.

## 4. Conclusion

In this study, biochar-based MnOx nanocomposites, specifically MnO_X_-CHBNC and MnO_X_-KBNC, were synthesized through the pyrolysis of CH and KL and used for the removal of MB from an aqueous solution. The highest MB removal efficiencies were observed when 25 g of each biomass was pretreated with 12.5 mmol of KMnO_4_ (at a ratio of 2 : 1 g·mmol^−1^) and pyrolyzed at 300°C for 1 h. The pristine biochars (CHB and KLB) and their corresponding MnO_X_-CHBNC and MnO_X_-KBNC possess porous amorphous structures. However, the MnO_x_-activated BNCs exhibit even more porous structures, higher specific areas, total pore volumes, and smaller pore sizes compared to the pristine biochars. Both the pristine and MnOx-activated biochars contain functional groups (O-H, C-H, C=C, and C-O) that may participate in adsorption through electrostatic interaction. The adsorption-desorption isotherm and BET analysis confirm the mesoporous structure of the adsorbents.

Various parameters that affect the adsorption efficiencies of MnO_X_-CHBNC and MnO_X_-KLBNC, such as solution pH, contact time, adsorbent dose, and initial concentration, were also investigated. The results indicate that the solution' pH has a negligible effect on the adsorption efficiencies of the adsorbents; therefore, a pH of 7.5 was chosen for the experiment. The optimal conditions for the other parameters were found to be a contact time of 60 min and an adsorbent dose of 0.15 g for MnO_X_-KLBNC and 0.2 g for MnO_X_-CHBNC.

Equilibrium adsorption studies reveal that both MnO_X_-CHBNC and MnO_X_-KLBNC fit well with the Langmuir isotherm model. Furthermore, the kinetic study results show that the adsorption mechanism of MB on both adsorbents follows the pseudo-second-order model. MnO_X_-KLBNC exhibited better stability compared to MnO_X_-CHBNC, with little change in the relative adsorption efficiency even after six cycles.

Overall, the advantages of MnOx-CHBNC and MnOx-KLBNC, such as their easy and fast production process, low cost, regeneration cycle, and environmental friendliness, make them suitable alternative adsorbents for MB removal. These findings can also serve as preliminary support for future studies aimed at improving the removal efficiency of MB using MnOx-based coffee husk and khat leftover biochar nanocomposite.

## Figures and Tables

**Table 1:** Preliminary experimental data for selection of adsorbents for removal of MB.

Adsorbent	KMnO_4_ (millimole)	Pyrolysis temperature (°C)	MB removal efficiency (%)
CHB_300_	0.0	300	39.08
CHB_400_	0.0	400	44.73
CHB_500_	0.0	500	80.78
**MnOx-CHBNC**_**300**_	**12.5**	**300**	**99.23**
MnOx-CHBNC_400_	12.5	400	89.96
MnOx-CHBNC_500_	12.5	500	96.10
MnOx-CHBNC_300_	25.0	300	97.20
MnOx-CHBNC_400_	25.0	400	96.95
MnOx-CHBNC_500_	25.0	500	93.99
MnOx-CHBNC_300_	50.0	300	84.40
MnOx-CHBNC_400_	50.0	400	95.33
MnOx-CHBNC_500_	50.0	500	95.51
MnOx-CHBNC_300_	75.0	300	66.65
MnOx-CHBNC_400_	75.0	400	97.46
MnOx-CHBNC_500_	75.0	500	86.38
KLB_300_	0.0	300	75.26
KLB_400_	0.0	400	76.41
KLB_500_	0.0	500	88.37
**MnOx-KLBNC**_**300**_	**12.5**	**300**	**98.01**
MnOx-KLBNC_400_	12.5	400	97.14
MnOx-KLBNC_500_	12.5	500	96.86
MnOx-KLBNC_300_	25.0	300	97.39
MnOx-KLBNC_400_	25.0	400	95.99
MnOx-KLBNC_500_	25.0	500	96.73
MnOx-KLBNC_300_	50.0	300	89.45
MnOx-KLBNC_400_	50.0	400	95.45
MnOx-KLBNC_500_	50.0	500	97.30
MnOx-KLBNC_300_	75.0	300	86.64
MnOx-KLBNC_400_	75.0	400	86.75
MnOx-KLBNC_500_	75.0	500	96.80

Bold values show that the materials selected are adsorbent.

**Table 2:** Langmuir and Freundlich isotherm constants for adsorption of MB.

Isotherm model	MnO_X_-CHBNC	MnO_X_-KLBCN
Langmuir	*y* = 0.0253*x* + 0.0442	*y* = 0.023*x* + 0.1281
*q*_*m*_	39.526	43.478
*K*_*L*_	0.572	1.000
*R*_*L*_	0.080	0.048
*R*^2^	0.999	0.991
Freundlich	*y* = 0.3421*x* + 2.4149	*y* = 0.3117*x* + 2.2975
*N*	2.923	3.208
*K*_*F*_	11.189	9.949
*R*^2^	0.679	0.812

**Table 3:** Constants of pseudo-first-order and pseudo-second-order adsorption kinetic models.

Adsorbent	Kinetic model	Initial MB concentration (mg·L^−1^)		
		20	100	300
MnO_X_-CHBNC	*q*_experimental_	1.976	9.972	29.329
	Pseudo-first-order			
	*q*_*e*_	1.048	6.309	21.092
	*K*_1_	3.44 × 10^−2^	5.20 × 10^−3^	1.10 × 10^−3^
	*R* ^2^	0.886	0.785	0.831
	Pseudo-second-order			
	*q*_*e*_	2.085	9.443	26.352
	*K*_2_	5.261 × 10^−2^	2.311 × 10^−3^	1.51 × 10^−2^
	*R* ^2^	0.998	0.976	0.999

MnO_X_-KLBNC	*q*_experimental_	2.629	13.240	33.104
	Pseudo-first-order			
	*q*_*e*_	1.546	3.428	25.280
	*K*_1_	4.20 × 10^−3^	9.40 × 10^−3^	1.60 × 10^−3^
	*R* ^2^	0.908	0.867	0.767
	Pseudo-second-order			
	*q*_*e*_	2.068	12.771	16.129
	*K*_2_	3.458 × 10^−2^	1.379 × 10^−2^	2.298 × 10^−3^
	*R* ^2^	0.997	0.999	0.974

**Table 4:** Comparison of the MB removal efficiency of some adsorbents from aqueous solution.

Adsorbent	*C*_o_ (mg·L^−1^)	*V*_o_ (mL)	*m* (g)	*q*_*m*_ (mg·g^−1^)	%*R*	Reference
Fe_3_O_4_/SDB nanocomposite	30.00	30.00	0.25	25.33	75.00	[3]
H_3_PO_4_-treated Beli biochar	10.00	50.00	0.30	12.32	90.00	[10]
Torrefied*-Acacia nilotica* biochar	50.00	50.00	2.00	158.30	ND	[10]
*Acacia nilotica* biochar	50.00	50.00	2.00	85.68	ND	[10]
Fe_3_O_4_@C core-shell comp	20.00	40.00	0.20	42.11	ND	[19]
NaOH-treated banana stem	25.00	50.00	0.80	0.47	96.59	[58]
NaOH-treated coffee husk	37.78	50.00	0.74	200.00	93.52	[65]
Fe_3_O_4_ nanopowder	20.00	25.00	2.00	25.54	99.69	[66]
Wheat straw-biochar	100.00	10.00	0.60	12.03	ND	[67]
KOH-activated CHBC	50.00	500.00	0.50	357.38	ND	[68]
MnO_x_-CHBNC	20.00	20.00	0.20	39.52	99.26	This study
MnO_x_-KLBNC	20.00	20.00	0.15	43.47	98.20	This study

## Data Availability

The necessary information is available from the corresponding author upon reasonable request.
